# The relationship between pregnancy and temporomandibular disorder (TMD) through diagnostic criteria for temporomandibular disorders (DC/TMD) axis II evaluation: a case-control cross-sectional study

**DOI:** 10.1186/s12903-024-04009-y

**Published:** 2024-03-16

**Authors:** Giuseppe Minervini, Maria Maddalena Marrapodi, Marco La Verde, Aida Meto, Yuliia Siurkel, Marco Cicciù, Diana Russo

**Affiliations:** 1 Saveetha Dental College and Hospitals, Saveetha Institute of Medical and Technical Sciences (SIMATS), Saveetha University, Chennai, Tamil Nadu India; 2 Multidisciplinary Department of Medical-Surgical and Odontostomatological Specialties, University of Campania “Luigi Vanvitelli”, 80121 Naples, Italy; 3 Department of Woman, Child and General and Specialized Surgery, Obstetrics and Gynecology Unit, University of Campania “Luigi Vanvitelli”, Largo Madonna delle Grazie 1, 80138 Naples, Italy; 4 Department of Dental Therapy, Faculty of Dental Medicine, University of Medicine, Tirana, Albania; 5 International European University School of Medicine, Akademika Hlushkova Ave, 42B, Kyiv, 03187 Ukraine; 6 Department of Biomedical and Surgical and Biomedical Sciences, Catania University, 95123 Catania, Italy

**Keywords:** Pregnancy, TMD, Bruxism, Temporomandibular disorders, TMD

## Abstract

**Introduction:**

This study focuses on temporomandibular disorders (TMDs), which affect the temporomandibular joint and related muscles and have multiple causes. Recent studies have examined the connection between menstrual cycles, estrogen levels, and TMDs, but results are inconsistent, highlighting the need for more research. The aim is to explore the prevalence of TMDs in pregnant women and consider how hormonal changes during pregnancy might influence these disorders.

**Methods:**

In this cross-sectional case-control study, we compared 32 pregnant women with 35 non-pregnant women. We evaluated several TMD-related factors such as pain levels, chronic pain classification, scores on the Jaw Functional Limitation Scale-20 and Oral Behaviors Checklist, and psychological health. We used various statistical methods including descriptive statistics, chi-square tests, linear regression, and adjustments for multiple comparisons to analyze the data.

**Results:**

Pregnant women showed different pain perceptions, generally reporting less pain and lower severity. Nonetheless, these differences were not uniform across all TMD-related measures. Linear regression did not find a consistent link between pregnancy and TMD scores, except for chronic pain grade, which was not significant after adjusting for multiple comparisons. There was a significant relationship between depression and TMD severity, emphasizing the need to consider mental health in TMD evaluations.

**Discussion:**

The findings suggest that pregnancy is neither a risk nor a protective factor for TMD. Differences in pain perception, functional status, and psychological health were observed in pregnant women but were not consistent for all TMD-related aspects. The role of estrogen in TMJ health and TMD risk is complex and requires further study. The research highlights the necessity of including mental health, especially depression, in TMD assessments. More comprehensive research with larger sample sizes is essential to better understand the connections between pregnancy, TMD, and hormones, aiming to improve TMD management in pregnant women and others.

**Supplementary Information:**

The online version contains supplementary material available at 10.1186/s12903-024-04009-y.

## Introduction

Temporomandibular disorders (TMDs) encompass a variety of conditions impacting the temporomandibular joint (TMJ) and its associated musculature [1, 2]. These conditions affect approximately 15% of adults and are most prevalent between the ages of 20 and 40, often manifesting as symptoms such as jaw discomfort, dysfunction, otalgia, facial pain, and headaches [2–5]. Their etiology is multifactorial, influenced by biological, environmental, social, and psychological factors [6, 7]. Given this complexity, the diagnosis and treatment of TMDs require a comprehensive approach that encompasses various diagnostic and therapeutic modalities. In the evaluation of TMDs, polysomnography serves as a crucial diagnostic tool, particularly in understanding the relationship between sleep disorders, such as sleep bruxism, and TMDs. Treatment approaches include physiotherapy, pharmacotherapy, psychotherapy, and the use of occlusal splints, addressing both the physical and psychological aspects of the disorder.

Recent investigations have delved into the potential correlation between varying menstrual states and estrogen levels with the incidence of TMDs [8, 9]. Specifically, numerous studies have explored the connection between pregnancy and TMD prevalence [8]. Estrogens, known for their role in regulating various physiological functions, including reproductive organ development, have been evaluated in relation to TMD [10–14]. The findings from these studies have reported conflicting findings, with some suggesting a possible association between elevated estrogen levels and heightened TMD risk, while others have found no such linkage [15–21]. Notably, estrogen levels can exert an influence on the structure and function of the TMJ [22–36].

Key insights have emerged from studies showing gender-related differences in estradiol concentration among individuals aged 15 to 45, implying a potential connection between estrogen levels and TMD, as well as other oral conditions like gingivitis [8]. However, epidemiological investigations investigating the relationship between estrogen levels and TMD prevalence and severity have yielded contradictory findings. On one hand, evidence indicates that TMDs predominantly affect women of childbearing age, implying a role for higher estrogen levels in increasing TMD risk [17, 21]. On the other hand, several epidemiological studies have identified an elevated risk of TMDs following menopause [18, 19, 37, 38].

From a pathogenic perspective, estrogen’s impact on cartilage has been noted, with certain studies demonstrating its protective effects on cartilage and bone [8]. Notably, estrogen treatment has resulted in a significant reduction in cartilage thickness and an increase in proteoglycan content [8]. These outcomes suggest that estrogen may play a part in maintaining TMJ cartilage integrity, potentially offering avenues for estrogen-based treatments for TMJ disorders.

Pregnant women often encounter an array of stressors that can potentially affect both maternal and fetal health. Chronic or severe stress during pregnancy has been associated with various adverse outcomes, including preterm birth, low birth weight, and developmental delays in offspring. Maternal stress may also lead to elevated levels of cortisol and other stress-related hormones, which can potentially traverse the placenta and impact fetal development. While a certain degree of stress during pregnancy is normal, healthcare providers should remain vigilant about potential stressors and offer support to help women manage stress levels, thus mitigating potential adverse effects on maternal and fetal health [35, 39–42].

Of note, the study by Radwan-Oczko et al. focuses on pregnant women's knowledge regarding oral health during pregnancy. It found that awareness about the importance of oral hygiene in this period is generally low. Only a minority of women had oral examinations before or during early pregnancy. The research also highlighted a positive correlation between frequent tooth brushing and higher birth weights, indicating the significance of oral care in pregnancy outcomes. This study underscores the need for better education and awareness among both pregnant women and healthcare providers about oral health during pregnancy.

TMDs can significantly affect an individual's quality of life and sleep. The pain severity associated with TMDs correlates with reduced life satisfaction and sleep quality. Understanding this relationship is crucial in developing comprehensive treatment plans that not only address the physical symptoms of TMDs but also consider their broader impact on an individual's well-being.

In light of this substantial body of evidence, our objective is to investigate the prevalence of TMDs among pregnant women.

## Methods

### Participants

In this case control cross-sectional study, we consecutively enrolled pregnant women and non-pregnant woman.

The study was conducted in accordance with the Declaration of Helsinki and this study was approved by the institute’s ethical committee of ALDENT UNIVERSITY [Protocol no. 846/2022; Date: 05/05/2022]The study was developed, and all subjects gave their written informed consent for inclusion before they participated in the study. Minors or illiterate are not involved in this study.

Pregnant Women Group: A total of 32 pregnant women formed this group. Comprehensive demographic data, including age, education, and pregnancy status, were gathered for each participant within this group.

Healthy Control Non-Pregnant Women Group: In parallel, a control group was assembled, encompassing 35 healthy non-pregnant women. Similar to the pregnant women group, detailed demographic information was collected for each participant within this control group, including age and educational background.

The study investigated various TMD-related variables using different scales and assessments:

- The Chronic Pain Grade (CPG) scale, also known as the Graded Chronic Pain Scale, is a tool used to assess and classify chronic pain in individuals. It was developed by researchers at the University of Washington in Seattle and is designed to provide a more comprehensive understanding of a person's chronic pain experience beyond just intensity. The CPG scale takes into account several dimensions of chronic pain, including:
  - Pain Intensity: This dimension assesses the severity of pain on a scale from 0 to 10, with 0 being no pain and 10 being the worst pain imaginable.
  - Pain-Related Disability: This dimension evaluates how much chronic pain interferes with a person's daily activities, including work, social life, and self-care.
  - Days in Pain: This dimension assesses how many days in the past six months a person has experienced significant pain.
  - Pain Intensity Variability: It considers whether the pain is relatively constant or if it fluctuates over time.
  - Pain Medication Use: This dimension examines the use of pain medication and whether it provides relief. Based on the scores in these dimensions, individuals can be categorized into one of five grades, ranging from Grade 0 to Grade IV.
- JPLS-20: The Jaw Functional Limitation Scale-20 subscales, including Mastication, Mobility, Communication, and Global, provide a comprehensive assessment of TMJ-related function and overall health status.
- Oral Behaviors Checklist (OBC): OBC was administered to ascertain the presence of oral behaviors associated with TMD. Scores on this checklist were compared between the pregnant women group and the control group.
- Psychological Well-being: The psychological well-being of participants was a key aspect of the study and was evaluated using the Patient Health Questionnaire-9 (PHQ-9) to assess depression levels. Furthermore, the Generalized Anxiety Disorder-7 (GAD-7) scale was employed to assess anxiety levels within the study cohort.

### Statistical analysis

A comprehensive suite of statistical analyses was undertaken to unveil relationships and discrepancies between pregnancy status (pregnant women vs. control group) and the diverse TMD-related variables. These analyses were detailed as follows:

An application of descriptive statistics was undertaken to provide an in-depth summary and comparative analysis of pain perception, chronic pain grade, JFLS scores, and OBC scores, quantifying the differences between pregnant women and the control group. Variables were treated as categorical variable based in the following standardized categories [43–45]. To compare the percentage of patients for each group between pregnant and non-pregnant women a chi square test was used.

To explore the intricate relationship between TMD scores and pregnancy status, a linear regression analysis was executed. This statistical technique allowed for the adjustment of potential confounding variables, including age, education, and depression (PHQ-9), providing a robust assessment of the associations. Independent variables were treated as continuous variables.

The rigorous application of corrections for multiple comparisons was paramount in this study when assessing the significance of the results. The most restrictive approach was used, Bonferroni correction. This approach was adopted to maintain the integrity of the Type I error rate control.

### Sample size calculation

To calculate the sample size for this study, we considered two groups: the pregnant group with an OBC score of 11 plus 9 standard deviations (SD) and the control group consisting of non-pregnant individuals with 24 participants. With a desired statistical power of 90% (1 - β), an alpha (α) level of 0.05, and an expected result of 27 participants per group, we employed a statistical formula or software to estimate the required sample size. It was determined that a sample size of 27 participants per group would be necessary to achieve a power of 90% at a significance level of 0.05, ensuring that the study is adequately powered to detect the anticipated effects or differences between the two groups.

## Results

### Population characteristics

Demographics are displayed in Table 1. The study sample consisted of 32 participants, primarily women, whose socio-demographic and clinical characteristics are the described in the following lines. The maternal age distribution was heterogeneous, with the majority falling between the ages of 30 to 35 years (40.6%) and 25 to 30 years (18.8%). The mean BMI prior to pregnancy was 25.68 ± 4.53 kg/m^2^. Comorbidities were prevalent, with 37.5% experiencing them in the first trimester, 40.6% in the second trimester, and 21.9% in the third trimester of pregnancy. Most participants had a high school education (50%), followed by secondary school (25%). A subset of the population reported tobacco use (18.8%). Comorbidities specific to pregnancy included gestational diabetes (6.3%) and gestational hypertension (3.1%), while no cases of preeclampsia were noted.

**Table 1:** Socio-demographic and clinical characteristics of the sample (*n* = 32)

	**Total sample (*n* 32)**
**Maternal age, years (%)**	
** 18-20 y**	1 (3.1)
** 20-25**	3 (9.4)
** 25-30**	6 (18.8)
** 30-35**	13 (40.6)
** 35-40**	6 (18.8)
** 40-45**	2 (6.3)
** 45-50**	1 (3.1)
**BMI prio to pregnancy, kg/m**^**2**^	
** Mean ± SD**	25.68 ± 4.53
** Mean ± SD**	
**Comorbidities, N (%):**	
** First trimester**	12 (37.5)
** Second trimester**	13 (40.6)
** Third trimester**	7 (21.9)
**Level of education, N (%):**	
** Primary School**	0 (0.0)
** Secondary School**	8 (25.0)
** High School**	16 (50.0)
** Degree**	8 (25.0)
**Tobacco use, yes, N (%):**	6 (18.8)
**Comorbidities, N (%):**	
** Gestational diabetes**	2 (6.3)
** Gestational hypertension**	1 (3.1)
** Preeclampsia**	0 (0.0)
**Conception, N (%):**	
** Spontaneous**	27 (84.4)
** In-vitro fertilization,**	5 (15.6)
**Marriage, N (%):**	23 (71.9)
**Celibacy, N (%):**	9 (28.1)
**Race, N (%):**	
** White**	31 (96.9)
** Black**	1 (3.1)

The majority of conceptions in the study were spontaneous, accounting for 84.4% of cases. Regarding the marital status of participants, 71.9% were married, while the remaining 28.1% were celibate. The racial composition was predominantly White (96.9%), with a small representation of Black individuals (3.1%). This diverse sample provides valuable insights into the characteristics of the population under study.

In the control group, the age distribution reveals a diverse composition. The majority of participants fall into two distinct age brackets, with 40.91% aged between 35 and 40 years and 31.82% in the 30 to 35-year range. Meanwhile, 18.18% of participants are in the 40 to 45-year category. Notably, there are no observations in the youngest age bracket of 15 to 20 years, and only a minor presence in the 20 to 25 and 25 to 30-year brackets, accounting for 4.55% each. Turning our attention to education levels, 46.88% of participants have completed university education, showcasing a substantial presence in this group. High school completion is also represented, with 13.64% of participants falling into this category. These results shed light on the age and educational profile of the control group, which will be vital for further analysis and interpretation of research findings. Therefore, patients in the control group were aged older and reported a higher educational level compared to the pregnant group.

**Fig. 1:**
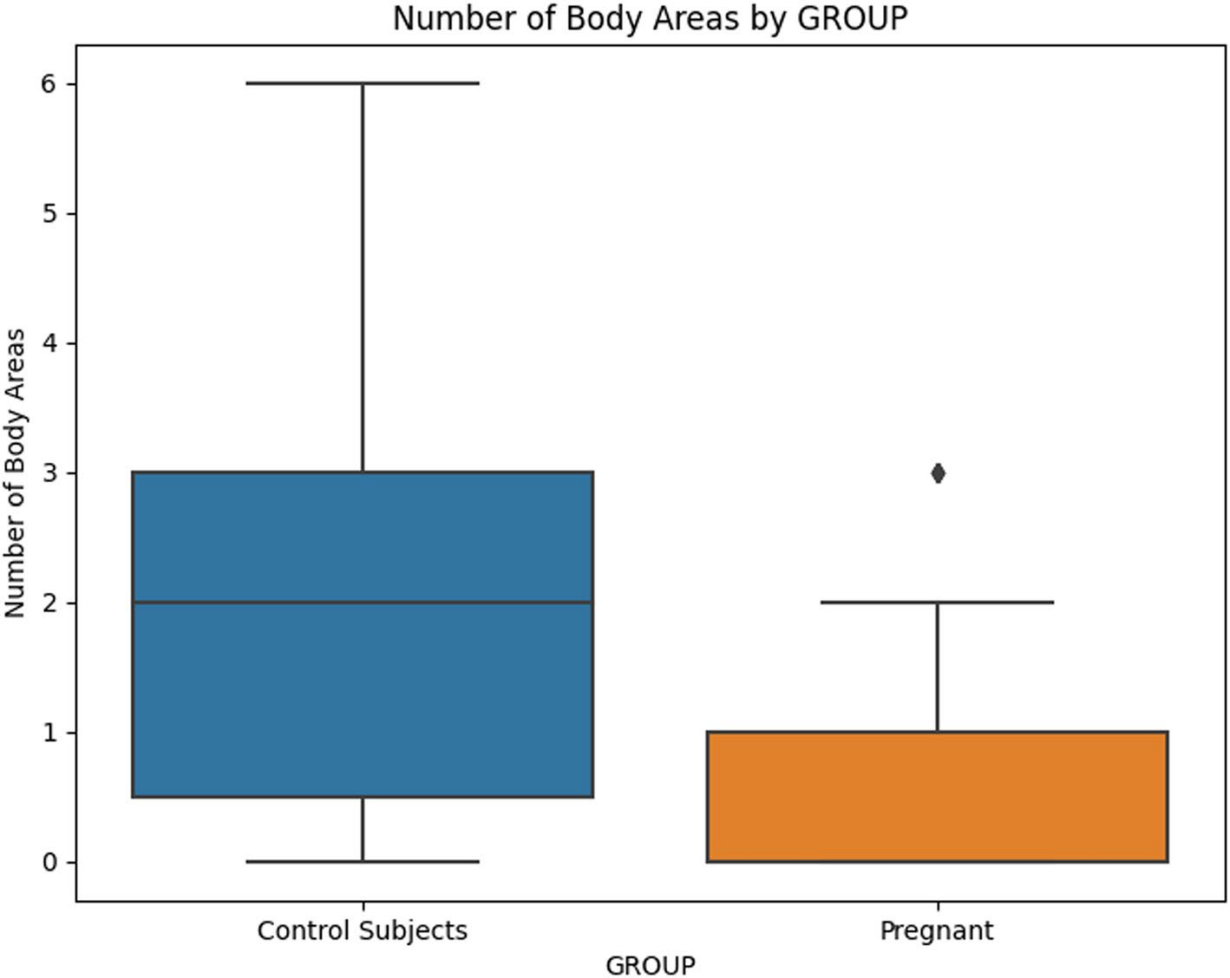
Mean value of "Number of Body Areas" for Pregnant and Control Subjects

Descriptive statistics reveal significant differences in number of body areas with pain between pregnant women and the control group (Table 2 and Fig. 1). In the study, a larger proportion of women in the pregnant group did not report pain (62.5%) compared to those in the control group (25.7%), as indicated in Table 2.However, there were no significant differences in pain intensity (Table 3) or pain interference (Table 4) between the two groups.

**Table 2:** Pain Drawing Total sample = 67

	**Pregnant (*n*=32)**	**Control Group (35)**	***p*-value**
**PAIN DRAWING, N(%)**			
** - 0**	20 (62.5)	9 (25.7)	0.05*
** - 1**	6 (18.8)	10 (29.4)	ns
** - 2**	2 (6.3)	7 (20.6)	ns
** - >2**	4 (12.5)	9 (26.5)	ns
**PAIN DRAWING, N(%)**			
** - None**	20 (62.5)	9 (25.7)	0.05*
** - Mild**	6 (18.8)	10 (29.4)	ns
** - Moderate**	2 (6.3)	7 (20.6)	ns
** - Severe**	4 (12.5)	9 (26.5)	ns

Chi-squared test

*ns* not significant

^*^P-value < 0.05

**Table 3:** GCPS, Characteristic Pain Intensity. Total sample = 67

	**Pregnant (*n*=32)**	**Control Group (35)**	***p*-value**
**Characteristic Pain Intensity, N(%)**			
** - 0**	27 (84.4)	16 (45.7)	Ns
** - 1-10**	3 (9.4)	4 (11.4)	Ns
** - 11-20**	1 (3.1)	2 (5.7)	Ns
** - 21-30**	0 (0.0)	2 (5.7)	Ns
** - 31-40**	0 (0.0)	4 (11.4)	Ns
** - 41-50**	0 (0.0)	2 (5.7)	Ns
** - 51-60**	0 (0.0)	2 (5.7)	Ns
** - 61-70**	1 (3.1)	1 (2.9)	Ns
** - 71-80**	0 (0.0)	1 (2.9)	Ns
** - 81-90**	0 (0.0)	1 (2.9)	Ns
** - 91-100**	0 (0.0)	1 (2.9)	Ns
**Characteristic Pain Intensity, N(%)**			
** - None**	27 (84.4)	16 (45.7)	ns
** - Low**	4 (12.5)	14 (40.0)	0.05*
** - High**	1 (3.1)	5 (14.3)	ns

Chi squared test

*ns* not significant

^*^P-value < 0.05

**Table 4:** GCPS - Interference Total sample = 67

	**Pregnant (*n*=32)**	**Control Group (35)**	***p*-value**
**Interference, N(%)**			
** - 0-29**	32 (100)	28 (80.0)	Ns
** - 30-49**	0 (0.0)	3 (8.6)	Ns
** - 50-69**	0 (0.0)	2 (5.7)	Ns
** - >70**	0 (0.0)	2 (5.7)	Ns
**Interference, N(%)**			
** - 0**	32 (100)	28 (80.0)	Ns
** - 1**	0 (0.0)	3 (8.6)	Ns
** - 2**	0 (0.0)	2 (5.7)	Ns
** - 3**	0 (0.0)	2 (5.7)	Ns

Chi-squared test

*ns* not significant

^*^P-value < 0.05

**Fig. 2:**
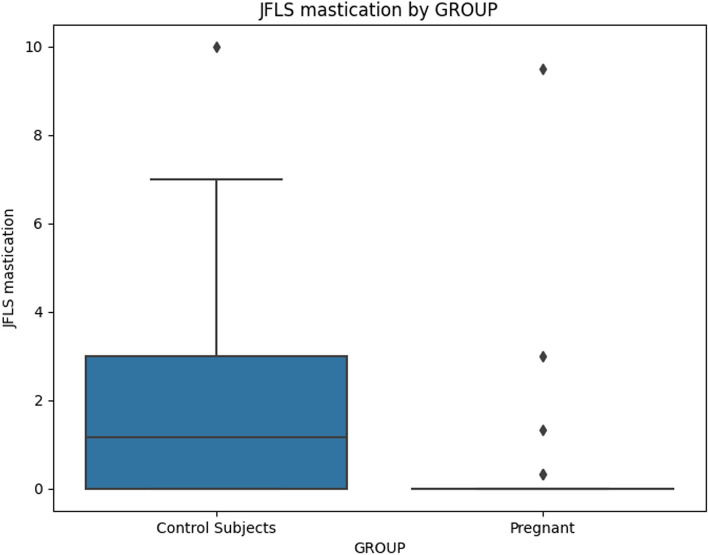
Mean value of "JFLS Mastication" for Pregnant and Control Subjects

**Fig. 3:**
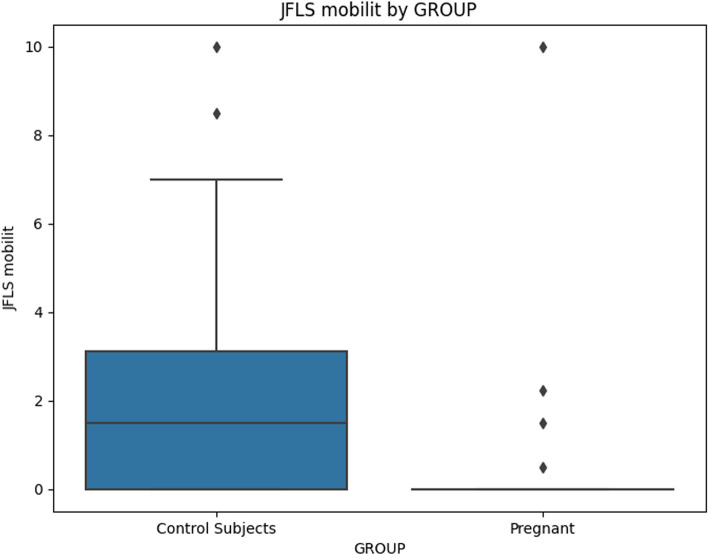
Mean value of "JFLS Mobility" for Pregnant and Control Subjects

**Fig. 4:**
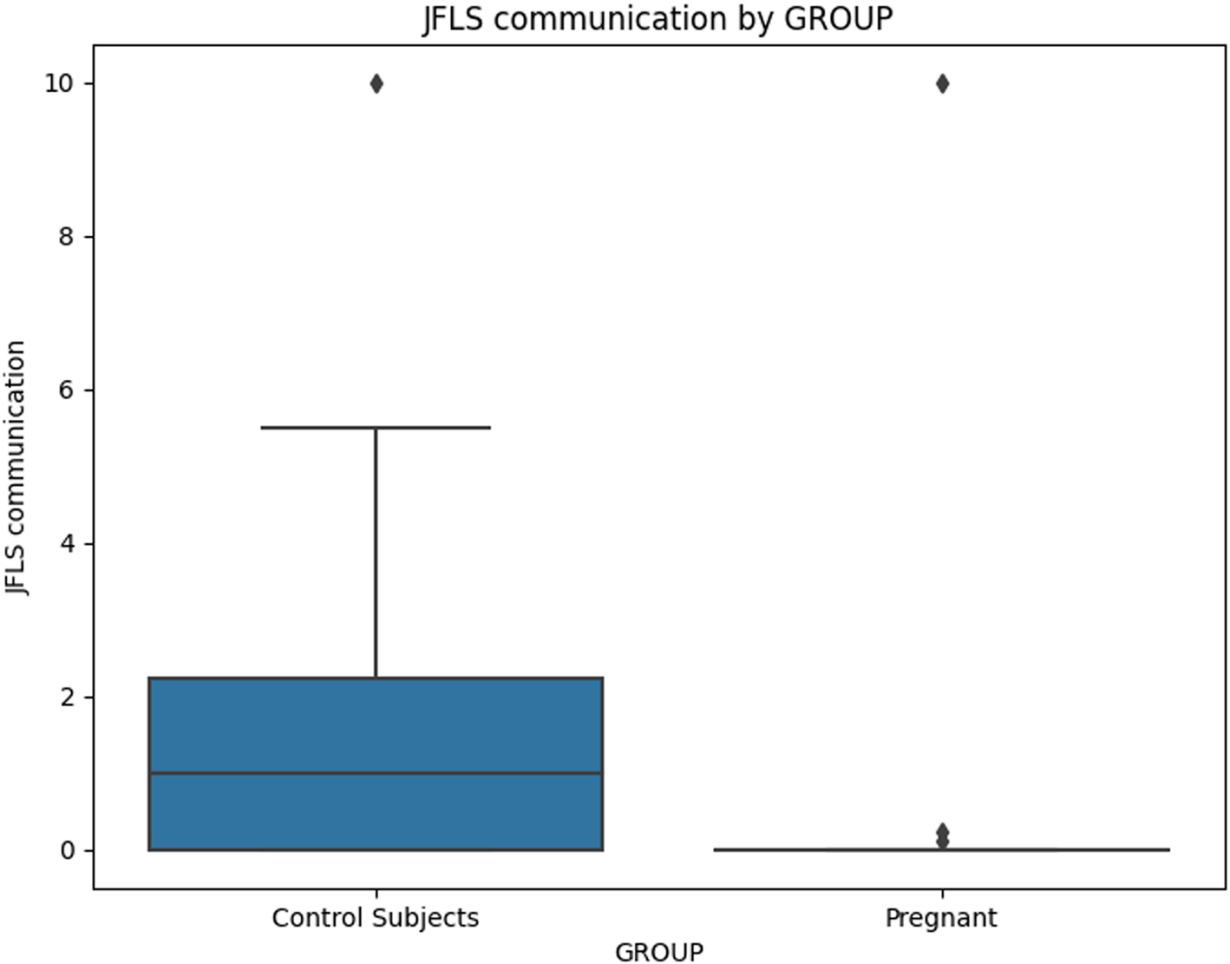
Mean value of "JFLS Communication" for Pregnant and Control Subjects

**Fig. 5:**
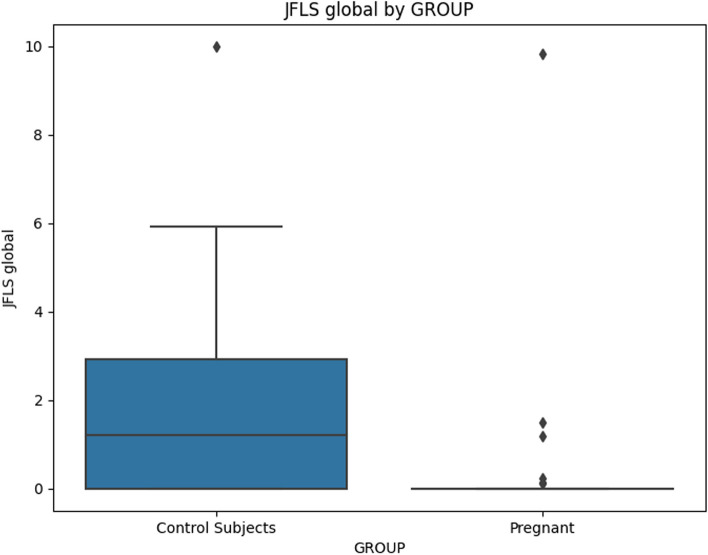
Mean value of "JFLS Global" for Pregnant and Control Subjects

When comparing chronic pain grade (Table 5), no significant differences emerged, indicating that both groups were similarly affected by chronic pain. The JFLS-20 scale (Table 6) indicated that pregnant women exhibited significantly lower scores across mastication, mobility, communication, and global functioning, implying lower dysfunction in these areas (Figs 2, 3, 4 and 5).

**Table 5:** GCPS - Chronic Pain Grade Total sample = 64

	**Pregnant (*n*=32)**	**Control Group (35)**	***p*-value**
**Chronic Pain Grade, N(%)**			
** - None**	26 (81.3)	31 (88.6)	ns
** - No Disability**	1 (3.1)	1 (2.9)	ns
** - Moderately Limiting**	3 (9.4)	2 (5.7)	ns
** - Severely Limiting**	2 (6.3)	1 (2.9)	ns
**Chronic Pain Grade, Grade N(%)**			
** - 0**	26 (81.3)	16 (45.7)	ns
** - I**	6 (18.8)	11 (31.4)	ns
** - II**	0 (0.0)	1 (2.9)	ns
** - III**	0 (0.0)	4 (11.4)	ns
** - IV**	0 (0.0)	3 (8.6)	ns

Chi-squared test

*ns* not significant

^*^P-value < 0.05

**Table 6:** JFLS-20. Total sample = 67

	**Pregnant (*n*=32)**	**Control Group (35)**	***p*-value**
**mastication** **Mean ± SD**	0.45 ± 1.74	2.21 ± 6.5	0.001*
**Mobilty** **Mean ± SD**	0.44 ± 1.80	2.43 ± 2.61	0.0006*
**Communication** **Mean ± SD**	0.32 ± 1.76	1.90 ± 2.27	0.0023*
**Global** **Mean ± SD**	0.40 ± 1.75	2.24 ± 2.51	0.009*

Indipendent simple T test

*ns* not significant

^*^P-value < 0.05

The study also examined psychological well-being. Pregnant women showed a lower prevalence of moderate depression (*p=*0.04) and mild depression (*p=*0.03) on the PHQ-9 scale (Table 7) and no difference in the prevalence of severe depression. However, no significant differences were observed in anxiety levels between the two groups using the GAD-7 scale (Table 8).

**Table 7:** PHQ-9 Depression Total sample = 67

	**Pregnant (*n*=32)**	**Control Group (34)**	***p*-value**
**PHQ-9 Depression, N(%)**			
** - 0-4**	17 (53.3)	6 (17.1)	0.03*
** - 5-9**	12 (37.5)	15 (42.9)	ns
** - 10-14**	2 (6.3)	10 (28.6)	0.04*
** - 15-19**	1 (3.1)	4 (11.4)	ns
** - 20-27**	0 (0.0)	0 (0.0)	ns
**PHQ-9 Depression, N(%)**			
** - None**	17 (53.3)	6 (17.1)	0.03*
** - Mild**	12 (37.5)	15 (42.9)	ns
** - Moderate**	2 (6.3)	10 (28.6)	0.04*
** - Mod-severe**	1 (3.1)	4 (11.4)	ns
** - Severe**	0 (0.0)	0 (0.0)	ns

Chi-squared test

*ns* not significant

^*^P-value < 0.05

**Table 8:** GAD-7, Anxiety. Total sample = 67

	**Pregnant (*n*=32)**	**Control Group (35)**	***p*-value**
**GAD-7, Anxiety, N(%)**			
** - 0-4**	15 (46.9)	12 (34.3)	0.49
** - 5-9**	13 (40.6)	18 (51.4)	0.59
** - 10-14**	3 (9.4)	3 (8.6)	0.91
** - 15-21**	1 (3.1)	2 (5.7)	0.62
**GAD-7, Anxiety, N(%)**			
** - None**	15 (46.9)	12 (34.3)	0.49
** - Mild**	13 (40.6)	18 (51.4)	0.59
** - Moderate**	3 (9.4)	3 (8.6)	0.91
** - Severe**	1 (3.1)	2 (5.7)	0.62

Chi squared test

*p*-value < 0.05

In Table 9, the comparison between pregnant and control groups reveals significant differences in high OBC Parafunction levels (*p=*0.001), with 3.1% pregnant and 45.7% control participants. No significant distinctions were found in other categories.

**Table 9:** OBC Parafunction. Total sample = 67

	**Pregnant (*n*=32)**	**Control Group (35)**	***p*-value**
**OBC Parafunction, N(%)**			
** - 0**	4 (12.5)	1 (2.9)	0.16
** - 1-24**	27 (84.4)	18 (51.4)	0.20
** - 25-84**	1 (3.1)	16 (45.7)	0.001*
**OBC Parafunction, N(%)**			
** - None**	4 (12.5)	1 (2.9)	0.16
** - Low**	27 (84.4)	18 (51.4)	0.20
** - High**	1 (3.1)	16 (45.7)	0.001*

Chi squared test

^*^p-value < 0.05

In summary, descriptive statistic showed that less pregnant women experienced pain in TMD region. They also exhibited lower dysfunction in various functional areas.

### Correlation analysis

Then, we investigated the correlation between the assessed TMD scores and pregnancy status, while adjusting for age, education, and depression (PHQ9). Linear regression analyses were performed for each TMD score measure separately and then the *p* value was adjusted for multiple comparisons.

### Number of body areas

The regression analysis for the number of body areas revealed no significant association between pregnancy status and the number of body areas affected (Coefficient = -0.1254, *p* = 0.697). However, depression (PHQ9) had a significant positive association with the number of body areas affected (Coefficient = 0.1144, *p* = 0.001), indicating that higher depression scores were associated with a greater number of affected body areas. This statistical significative difference remained consistent after multiple comparison correction (*p=*0.009).

### Pain intensity

For pain intensity, the regression analysis showed no statistically significant association between pregnancy status and pain intensity (Coefficient = -0.4409, *p* = 0.093). Additionally, none of the covariates (age, education, and depression) had significant associations with pain intensity.

### Interference

The analysis of interference scores revealed no significant relationship between pregnancy status and interference caused by TMD (Coefficient = -0.2080, *p* = 0.279). None of the covariates showed significant associations with interference.

### Chronic pain grade

The regression analysis for chronic pain grade demonstrated a significant negative association between pregnancy status and chronic pain grade (Coefficient = -0.6734, *p* = 0.032), indicating that pregnant individuals had lower chronic pain grades compared to non-pregnant individuals. However, after correction for multiple comparisons the significance was lost. None of the other covariates showed significant associations with chronic pain grade.

### JFLS mastication

The analysis of JFLS Mastication scores showed a marginally significant negative association with pregnancy status (Coefficient = -1.4679, *p* = 0.055). Age, education, and depression did not have significant associations with JFLS Mastication scores.

### JFLS mobility

For JFLS Mobility scores, the regression analysis revealed a significant negative association with pregnancy status (Coefficient = -1.7803, *p* = 0.024), indicating that pregnant individuals had lower JFLS Mobility scores. However, after correction for multiple comparisons the significance was lost. None of the other covariates showed significant associations with JFLS Mobility.

### JFLS communication

The analysis of JFLS Communication scores did not yield any significant association with pregnancy status or any of the covariates (age, education, and depression).

### JFLS global

The regression analysis for JFLS Global scores demonstrated a significant negative association with pregnancy status (Coefficient = -1.6081, *p* = 0.031), indicating that pregnant individuals had lower JFLS Global scores. However, even in this case, after correction for multiple comparisons the significance was lost. None of the other covariates showed significant associations with JFLS Global scores.

#### OBC

The analysis of the OBC scores revealed a significant negative association with pregnancy status (Coefficient = -7.2970, *p* = 0.047), indicating that pregnant individuals had lower OBC scores. However, after correction for multiple comparisons the significance was lost. Additionally, depression (PHQ9) was significantly associated with OBC scores (Coefficient = 1.4353, *p* < 0.001), indicating that higher depression scores were related to higher OBC scores. This latter association remained consistent after Bonferroni correction.

Overall, these results suggest that pregnancy is neither a risk factor nor a protective one. However, our results indicate a negative trend between pregnancy status and numerous scales exploring TMD health. Additionally, depression (PHQ9) appears to be a significant predictor of several TMD score measures, highlighting the importance of considering mental health factors in the assessment of TMD.

## Discussion

In this study investigating the relationship between pregnancy and TMD, a wealth of valuable insights has emerged. The study encompassed pregnant women and healthy control non-pregnant women, with an array of TMD-related variables meticulously assessed and analyzed [46–50].

Descriptive statistics demonstrated that pregnant women exhibited significant differences in pain perception compared to the control group, with a higher proportion of pregnant women not reporting pain and experiencing lower pain severity [51–53]. However, no significant disparities were observed in characteristic pain intensity or pain interference between the two groups. The study employed the JFLS to evaluate various dimensions of functional status, including mastication, mobility, communication, and global functioning. The analysis indicated that pregnant women displayed lower dysfunction in these areas, as evidenced by significantly lower scores on the JFLS scale. Nevertheless, it's essential to note that after correcting for multiple comparisons, the significance was lost in some cases. Psychological well-being, a critical component of TMD assessment, was explored using the PHQ-9 and the GAD-7 scale. Pregnant women demonstrated a lower prevalence of moderate and mild depression on the PHQ-9 scale, highlighting potential differences in mental health between the two groups. However, no significant differences in anxiety levels were observed using the GAD-7 scale. Linear regression analyses were performed to delve deeper into the relationship between TMD scores and pregnancy status while adjusting for covariates such as age, education, and depression (PHQ-9) [54]. The results indicated that pregnancy status was not consistently associated with TMD-related variables, except for chronic pain grade, where pregnant individuals exhibited lower grades compared to non-pregnant individuals. However, this significance was lost after correction for multiple comparisons. An important finding from the study is the significant association between depression (PHQ-9) and several TMD-related measures. Higher depression scores were related to increased TMD severity, particularly as indicated by the OBC scores.

Overall, this study results suggest that pregnancy itself is neither a definitive risk factor nor a protective factor for TMD. While pregnant women demonstrated differences in pain perception, functional status, and psychological well-being, these differences were not consistent across all TMD-related variables, and some significance was lost after correcting for multiple comparisons. The findings do, however, indicate a negative trend between pregnancy status and certain TMD health scales, hinting at potential associations worth exploring further. Furthermore, the study underscores the significance of considering mental health factors, particularly depression (PHQ-9), in the assessment of TMD. Elevated depression scores were associated with increased TMD severity, emphasizing the need for a holistic approach to TMD evaluation that incorporates mental health assessments.

Estrogen, a hormone with a pivotal role in numerous physiological processes, including bone and joint health, has garnered attention for its influence on the TMJ [8]. Recent investigations have illuminated the potential link between estrogen levels and TMDs [9]. This hormone's intricate involvement in the TMJ is multifaceted. The TMJ, a complex joint encompassing muscles, ligaments, nerves, and cartilage, faces the modulatory effects of estrogen. Studies have revealed that estrogen deficiency is associated with an increased risk of TMD. Estrogen, it seems, exerts its influence on the TMJ by regulating the expression of crucial proteins, such as collagen and elastin [24, 25]. Additionally, estrogen impacts the production of prostaglandins, hormones vital for cartilage balance [55]. Furthermore, estrogen's role extends to bone health, with its deficiency linked to a heightened risk of osteoporosis, characterized by fragile bones [56]. Notably, estrogen contributes to the strength of TMJ-supporting muscles like the masseter and temporalis [57]. From a pathogenic perspective, estrogen appears to play a role in TMD development [20]. While some evidence suggests that higher estrogen levels in women of childbearing age may increase TMD risk, other studies point to an elevated risk post-menopause [20]. Reduced collagen production due to estrogen deficiency may lead to joint instability, potentially contributing to TMD. Moreover, estrogen deficiency has been linked to heightened pain sensitivity. Interestingly, estrogen also emerges as a player in TMD treatment [20]. Research indicates that estrogen replacement therapy can alleviate pain, enhance function, and expedite healing post-TMD treatment [8]. In sum, estrogen occupies a significant role in both the maintenance and treatment of TMJ disorders. While pregnancy introduces a range of physiological changes in women, including hormonal fluctuations, increased weight, and postural adjustments, it's vital to recognize that these changes can impact the TMJ. Physiologically, hormonal shifts during pregnancy may cause structural alterations, such as swelling, in the TMJ and its surrounding structures [8]. This swelling can restrict jaw movement due to reduced space [21, 58]. Additionally, pregnancy can weaken the muscles and ligaments supporting the jaw, potentially leading to reduced jaw strength and mobility. Studies have offered contrasting insights into pregnancy's influence on TMJ health [59, 60]. Some suggest an increase in TMJ laxity and decreased musculoskeletal orofacial pain during pregnancy [21]. Conversely, other research associated pregnancy with systemic hypermobility, unrelated to TMJ hypermobility [14]. Additionally, experimental pain studies indicate that high levels of estrogen and progesterone possess antinociceptive properties [61]. In a recent systematic review [62], the prevalence of TMD in pregnant women was explored, with findings indicating that it does not significantly differ from non-pregnant childbearing women. However, variations in diagnostic criteria may have contributed to the diversity in reported prevalence rates across studies. Notably, studies applying the RDC/TMD provide more robust evidence, strengthening the assertion that pregnancy is not a significant risk factor for TMD.

In the context of our discussion, it's important to note the relevance of Cone-Beam Computed Tomography (CBCT) in diagnosing degenerative changes in the temporomandibular joint (TMJ). A recent study by Görürgöz et al. (ref) conducted a multicenter CBCT investigation, focusing on degenerative changes in the mandibular condyle and their relation to TMJ space, gender, and age. This research emphasized that CBCT offers multiplanar views of TMJ bone components and pathologies without distortion, with condylar flattening being a frequently observed degenerative change. However, it's crucial to consider that the use of CBCT is limited during pregnancy due to radiation concerns.

In conclusion, in line with the most recent studies, this study highlights the complexity of this association and strength the assumption that pregnancy is neither a risk factor nor a protective one. Further research, with larger sample sizes and more extensive investigations, is warranted to elucidate the nuanced interplay between pregnancy, TMD, and mental health factors. Such endeavors can enhance our understanding and ultimately contribute to improved care and management of TMD in pregnant women and beyond.

### Supplementary Information

**Supplementary Material 1. **

## Data Availability

The data will be available on reasonable request from the corresponding author.
